# SliDL: A toolbox for processing whole-slide images in deep learning

**DOI:** 10.1371/journal.pone.0289499

**Published:** 2023-08-07

**Authors:** Adam G. Berman, William R. Orchard, Marcel Gehrung, Florian Markowetz

**Affiliations:** Cancer Research UK Cambridge Institute, University of Cambridge, Cambridge, United Kingdom; University of A Coruña, SPAIN

## Abstract

The inspection of stained tissue slides by pathologists is essential for the early detection, diagnosis and monitoring of disease. Recently, deep learning methods for the analysis of whole-slide images (WSIs) have shown excellent performance on these tasks, and have the potential to substantially reduce the workload of pathologists. However, WSIs present a number of unique challenges for analysis, requiring special consideration of image annotations, slide and image artefacts, and evaluation of WSI-trained model performance. Here we introduce SliDL, a Python library for performing pre- and post-processing of WSIs. SliDL makes WSI data handling easy, allowing users to perform essential processing tasks in a few simple lines of code, bridging the gap between standard image analysis and WSI analysis. We introduce each of the main functionalities within SliDL: from annotation and tile extraction to tissue detection and model evaluation. We also provide ‘code snippets’ to guide the user in running SliDL. SliDL has been designed to interact with PyTorch, one of the most widely used deep learning libraries, allowing seamless integration into deep learning workflows. By providing a framework in which deep learning methods for WSI analysis can be developed and applied, SliDL aims to increase the accessibility of an important application of deep learning.

## Introduction

In histopathology, tissue biopsies are fixed, embedded, sectioned, stained, and placed on a glass slide before being examined under a microscope. Examination of tissue slides to identify pathologically relevant features has been an essential tool for early detection, diagnosis and disease monitoring in medical practice and research for decades. Pathological features can be anything from the presence or absence of certain cell types or populations, changes in cellular or nuclear morphology, changes in the arrangement of cells in a tissue, to changes in the intensity of certain tissue stains. Until recently only expert pathologists have been able to perform this task, requiring years of training, and with individual slides often having to be evaluated by multiple pathologists before a judgement can be made [1]. However, with a shift towards digitisation in pathology, tissue-slides are now routinely scanned to produce high-resolution whole-slide images (WSIs). Such images are amenable to automated image analysis and in the last decade the field has undergone a revolution. Deep learning methods for image analysis have shown excellent performance on diagnostic tasks [1–3], rivalling that of pathologists and further stimulating efforts to digitise glass slides.

Pathologists have high inter-observer concordance rates on some diagnostic tasks, but in others they frequently disagree [4]. This is compounded by high workload, necessitating rapid screening of individual cases, increasing the risk of introducing diagnostic errors [5]. Deep learning methods are fast, often requiring only a few minutes to evaluate a slide, and give consistent evaluations. Thus, deep learning has the potential to substantially reduce the workload of pathologists, improve the inter-observer concordance rates and accelerate the evaluation of tissue-slides. The application of deep learning to pathological datasets is therefore a quickly growing field, as researchers apply the latest advances in machine learning, such as GANs [6, 7] and transformers [8, 9], to whole slide image problems [10, 11].

Despite this potential, deep learning based approaches have not yet seen widespread uptake in medical practice. This is in part due to a lack of an accessible framework in which WSI neural network implementations are developed and applied, meaning that individual researchers often must re-implement their own pre- and post-processing pipelines in-house for each new histopathology task. Furthermore, successful implementation of deep learning to WSI analysis requires careful consideration of model hyperparameters, slide and image artefacts and data augmentation beyond those encountered in standard image analysis, and thus application of the latest advances in computer vision to WSI analysis is hampered without a framework for streamlined WSI processing into which such advances can be incorporated.

**Fig 1:**
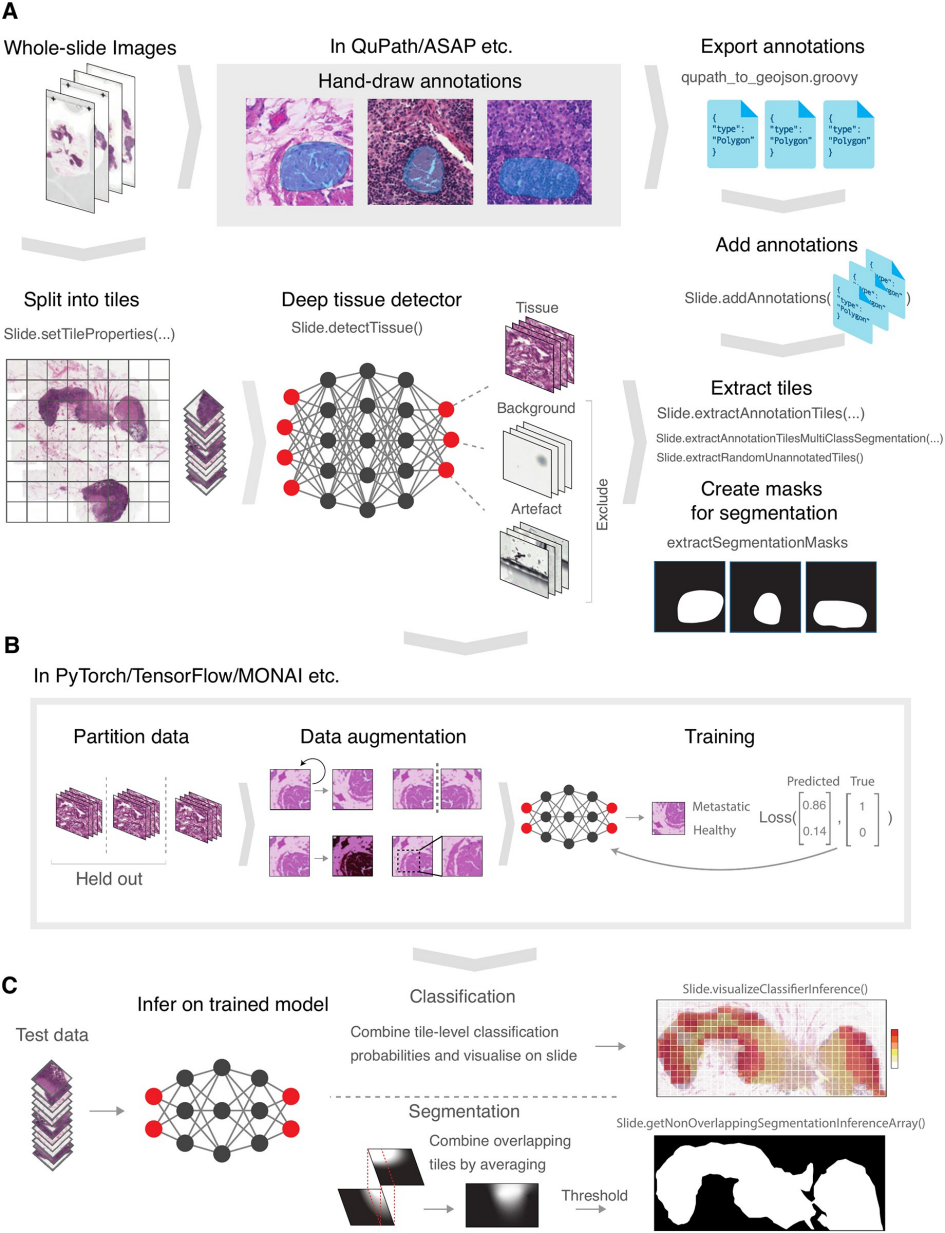
A deep learning pipeline with SliDL. **(A)** Creating SliDL Slide objects, exporting and adding annotations, and extracting tiles and segmentation masks. **(B)** Data partitioning, data augmentation, and model training. **(C)** Inferring on the trained model and stitching together overlapping segmentation results (if required).

Here we introduce SliDL, a new Python library for performing pre- and post-processing of WSI data. SliDL simplifies and streamlines many of the steps required to tackle the unique challenges posed by WSIs. This includes, but is not limited to, detection of tissue, slide and tissue artefacts and background in WSIs, easy implementation of alternative tiling strategies, automatic generation of binary and multi-class segmentation masks from digital annotations, and utility functions for visualisation and evaluation of model outputs (see Fig 1 and S1 Table for an overview of the main functionalities in SliDL). Although other tools exist which provide some of the same functionalities for pre-processing, SliDL is unique in its comprehensive support for annotation handling (see Related methods). By simplifying and streamlining these steps, SliDL aims to empower researchers in the clinical sciences to accelerate the application of deep learning to both existing and newly generated WSI data, so that the latest innovations in digital pathology can reach the clinic sooner.

SliDL therefore takes into account all of eccentricities of the WSI data type. For example, their large size makes it is necessary to break up WSIs into ‘tiles’ before they can be analysed by contemporary deep learning architectures (see Tiling). Tiling, however, introduces further difficulties as WSIs are often labelled (e.g. cancerous or non-cancerous) at the slide level, not at the tile level, and so a deep learning approach must be adopted which accounts for how tiles inherit labels from slides (see Annotation). Furthermore, WSIs can contain unique artefacts introduced during slide preparation and imaging, which are not found in other image analysis settings, such as pen marks left by the pathologists reviewing them, or cracks and bubbles in the slide. All of these artefacts must be removed or accounted for when training a deep learning model (see Deep tissue detector). SliDL includes easy-to-use functions to both perform tiling and to filter out artefact and background slides using a built-in deep neural network.

SliDL has been designed to interact with popular deep learning library PyTorch [12], allowing it to be seamlessly incorporated into deep learning workflows. By tackling the unique challenges posed by WSIs, SliDL can help to translate deep learning methods into the clinic more easily, providing a broad method to replace *ad hoc* solutions, and an accessible entry point to applying deep learning to WSIs for machine learning researchers unfamiliar with the nuances of pathology slides. SliDL is available from https://github.com/markowetzlab/slidl, documentation is available at https://slidl.readthedocs.io/en/latest/, and a full tutorial and example SliDL workflow is available at https://github.com/markowetzlab/slidl-tutorial, which trains, infers, and evaluates classification and segmentation models built from a balanced dataset of nine lymph node node section WSIs containing breast cancer metastases, and nine without metastases. The WSIs come from the publicly-available CAMELYON16 dataset [13].

SliDL was motivated by a perceived gap in existing tools comprising a number of features we see as crucial to the application of deep learning to histopathological tasks, which we therefore implemented and included in SliDL. Among these are its in-built, robust, benchmarked deep tissue detector, its numerous functions for reading in and extracting tiles from digital annotations, its capacity to generate tile-level segmentation masks, and its capacity to perform inference and compute performance metrics (see Distinct advantages of SliDL). All of these features and many more are made immediately accessible to novice and expert digital pathologists: SliDL is capable of shortening hundreds of lines of code requiring in-depth knowledge of image analysis into five to fifteen idiomatic lines which can be understood and implemented quickly and easily.

In this article we describe each of the major functionalities of SliDL in the order of their application in a typical deep learning pipeline. First showing how WSI data is handled within SliDL and how to import WSIs (Handling whole-slide images), implement different tiling strategies (Tiling). Then, we move on to how to apply the ‘deep tissue detector’ to detect tissue and remove background and artefacts from slides (Deep tissue detector). Next, we show how SliDL enables handling of digital annotations and the extraction of tiles and their corresponding segmentation masks (Annotation), before finally demonstrating how the library can be used to streamline various aspects of model training (Training), inference (Inference) and evaluation (Evaluating model performance). In each section, ‘code snippets’ are provided giving guidance on how SliDL should be run (see S2 Table for a table detailing each of the functions displayed in code snippets below and defining their arguments).

## Implementation

### Handling whole-slide images

When glass slides are digitised by digital whole-slide image scanners, high-resolution images are taken at multiple magnifications. WSIs therefore have a pyramidal data structure, with the images taken at each magnification each forming a ‘layer’ of the WSI. The maximum magnification of these images is frequently 200X (by convention called ‘20X’, due to scanning being performed using a 20X objective lens at 10X magnification) or 400X (by convention ‘40X’, using a 40X objective lens at 10X magnification) [14].

SliDL uses the Pyvips library for reading WSIs [15], and so supports a wide range of formats, including NDPI and pyramidal TIFF, including all OpenSlide formats, and those which are loaded via ImageMagick or GraphicsMagick such as DICOM [14]. After importing SliDL, WSIs are instantiated as Slide objects by calling the Slide class on the file path to the WSI and specifying which layer you would like to access with the level argument.

**Code Listing 1**. **Import SliDL and initialise a Slide object with a path to a WSI**


from slidl.slide import Slide
slidl_slide = Slide(path_to_wsi, level=0)


It is through Slide objects that the user interacts with their data and performs the pre- and post-processing steps described in the following sections.

During pre- and post-processing, SliDL Slide objects are generally modified in-place, with new information being added to an internal dictionary (called the ‘tile dictionary’, see Tiling below). It is therefore important that users save Slide objects after performing an operation on them, particularly time-intensive operations such as applying the deep tissue detector (see Deep tissue detector below). By doing this, the user does not have to wait for expensive functions to complete more than once.

Saving Slide is achieved by using the Slide.save() method, preserving the entire Slide object in its current state to a .pml file in the directory specified by the folder argument using lossless compression.

**Code Listing 2**. **Save a Slide object**


slidl_slide.save(folder='path/to/folder')


To reload a Slide object which has been saved, simply set the first argument of the Slide initialiser to the Slide object rather than to a WSI.

**Code Listing 3**. **Reload a saved Slide object**


slidl_slide = Slide('/path/to/folder/slidl_slide.pml', level=0)


### Tiling

WSI images are very large; for example, an image scanned at 40X objective power of a 20 x 20 mm sample of tissue has 80,000 x 80,000 pixels; at standard 24-bit colour this would produce a flat image 19.2GB in size. Current neural network architectures are unable to process images of this size in one go. Thus, WSIs are broken up into ‘tiles’ or ‘patches’ upon which the model is trained: small square regions of the original image, typically 32 to 1000 pixels in height. Tiles can be chosen with or without overlap with neighbouring tiles. Choice of tile dimensions and overlap are some of the most important hyperparameters to choose when analysing WSIs [16].

In SliDL, tile dimensions and overlap are chosen by calling the Slide.setTileProperties() method, and setting the tileSize and tileOverlap arguments, enabling users to easily experiment with different tiling strategies. Here one can also specify how tiles should be stored, and how they will be accessed during training.

**Code Listing 4**. **Set the tile properties in a Slide**


slidl_slide.setTileProperties(tileSize=500, tileOverlap=0, unit='px')


By calling Slide.extractAnnotationTiles() or Slide.extractRandomUnannotatedTiles(), one can extract and store each tile in individual images files in advance of training or inferring. SliDL will automatically store tile image files according to their class (see Annotation) and slide of origin in a directory structure appropriate for use with PyTorch see S1 Fig).

Although functional, storing each individual tile image file may pose data storage issues. SliDL stores the coordinates of each tile rather than the tile image itself, accessible with Slide.getTile() using the tile address as argument (all tile addresses can be iterated over with Slide.iterateTiles()), making it easy to build a dataset such that tiles are accessed on-the-fly, saving the need to extract each tile to an image file in situations where this would be too memory intensive.

**Code Listing 5**. **Iterate through the tiles in a Slide**


for tile_coords in Slide.iterateTiles():
    pyvips_tile_image = slidl_slide.getTile(tile_coords)


Apart from being substantially less disk memory intensive, this approach also makes it easier to experiment with different tiling strategies without having to re-extract tiles for each combination of tile size and overlap. The trade off is that on-the-fly tile accession approaches are typically much slower to train with, so are not recommended except in datasets where tiles number in the hundreds of thousands or millions and disk memory for these tile images is not available.

### Deep tissue detector

WSIs can contain unique artefacts introduced during slide preparation and imaging which are not found in other image analysis settings. Tissue may tear and fold during slide preparation, the image may be unevenly illuminated or stained, and parts of the image may be out of focus. Tissue slides also often contain pen marks left by the pathologists reviewing them, and may contain cracks and bubbles. Left unaccounted for, such artefacts can have severe detrimental effects on a deep learning model. For instance, pen marks are often left by pathologists to indicate the presence of a pathological feature of interest, such as the presence of cancerous cells. If not removed, a deep learning model may simply learn to recognise the presence of a pen mark in slides containing cancer, and thereby be completely inapplicable in medical practice where no such annotation will be available. Beyond artefacts, WSIs will typically contain large portions of background, i.e. regions without any tissue, which do not contain any pathologically relevant information. After tiling your WSI, tiles which contain artefacts or which simply display background should therefore be removed speed up the training process and potentially improve performance.

**Fig 2:**
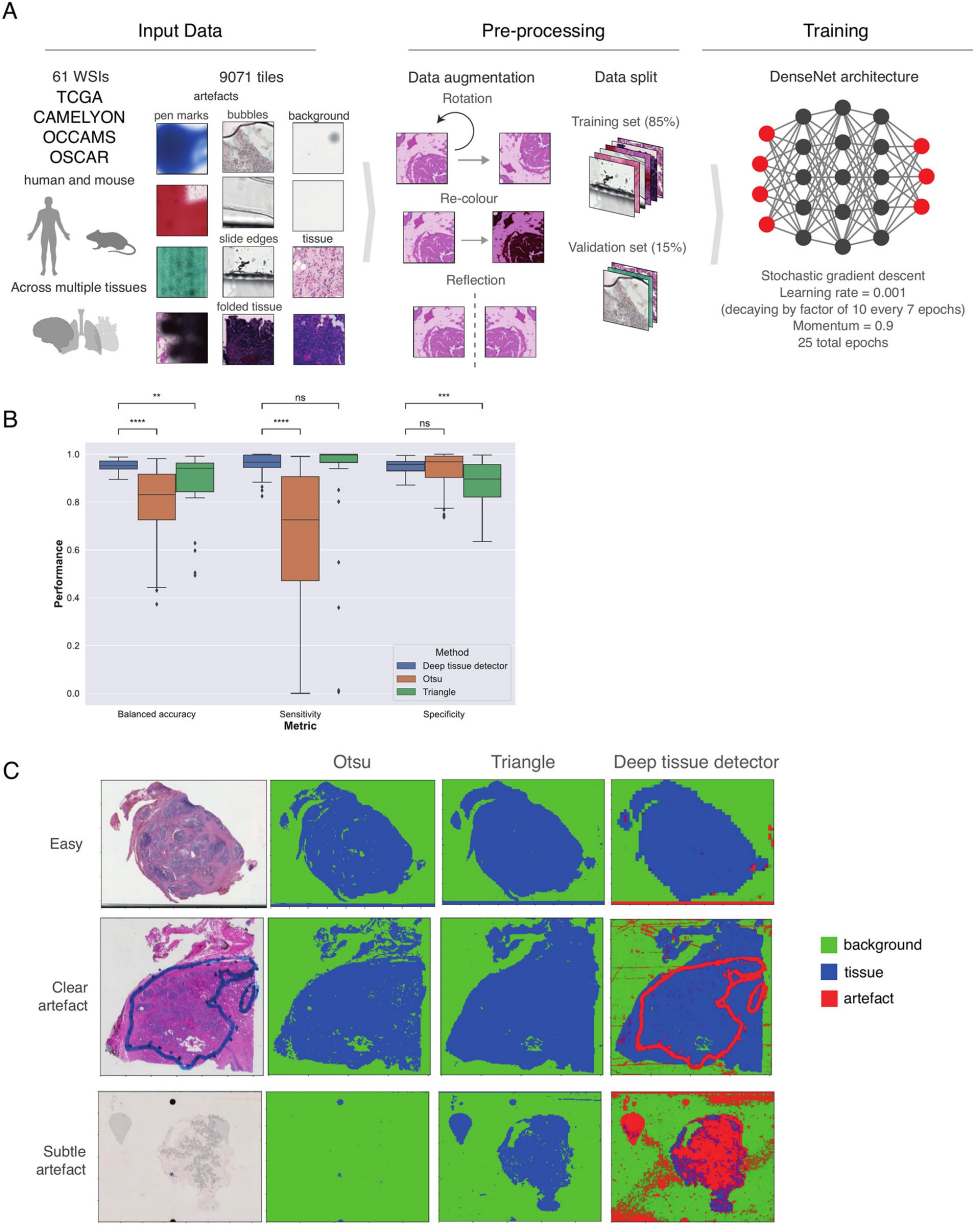
Tissue, artefact, and background detection with SliDL. **(A)** Tiling, augmenting, and training a DenseNet CNN to classify tissue, artefact, and background regions on WSIs from a robust dataset representing multiple tissue and species types. This already-trained deep tissue detector can be applied to any SliDL Slide object with SliDL’s Slide.detectTissue() function. **(B)** Comparison of classical foreground methods to the Deep tissue detector. All tests performed are two-tailed Wilcoxon signed-rank tests (*n* = 36). All *P* values are Benjamini-Hochberg adjusted, *P < 0.05, **P < 0.01, ***P < 0.001. **(C)** Three representative sample slides on which benchmarking was performed. The top row displaying a case where tissue and background are easily distinguished and all three approaches perform well. The middle row displaying a case where a clear pen mark artefact is incorrectly identified as tissue by the two classical approaches, indicating that artefact removal pre-processing is necessary, but the deep tissue detector has automatically performed both artefact and tissue detection. The bottom row displaying where a large bubble artefact obscures much of the tissue. Otsu classifies almost the entire slide as background, Triangle does not exclude tissue obscured by the artefact, and the deep tissue detector successfully identifies both.

SliDL provides a built-in deep tissue detector: a DenseNet [17] convolutional neural network architecture (see Convolutional neural networks) trained to classify tiles as either ‘artefact’, ‘background’, or ‘tissue’. SliDL’s deep tissue detector was trained using 9,071 tiles extracted from 393 individual annotations from 61 WSIs scanned across a variety of machines, time periods, and tissue types, and two different species to account for a broad range of the variation of WSI artefacts (including pen marks, folded or torn tissue, slide bubbles, cracks, blurred or out-of-focus regions, uneven illumination, aberrant staining and other marks), background, and tissue appearances (Fig 2A). An imbalanced dataset sampler [18] was used to ensure that during training, the model was exposed to equal numbers of artefact, background, and tissue tiles. The deep tissue detector is applied by calling the Slide.detectTissue() method, enabling robust detection of tissue tiles at any level of the WSI pyramid desired. SliDL then saves the output probabilities that each tile belongs to each of the three classes internally for each tile in the Slide object.

**Code Listing 6**. **Apply the deep tissue detector to a Slide**


slidl_slide.detectTissue(tissueDetectionLevel=1,
                            tissueDetectionTileSize=512,
                            tissueDetectionTileOverlap=0,
                            tissueDetectionUpsampleFactor=4,
                            batchSize=20,
                            numWorkers=16)


There may be applications where the artefacts encountered are not well covered by the deep tissue detector in SliDL, and thus one should always review examples of its output to verify that the detector is behaving as expected using SliDL’s Slide.visualizeTissueDetection() function (Fig 2C).

**Code Listing 7**. **Verify the results of the deep tissue detector**


slidl_slide.visualizeTissueDetection(fileName='tissue_detection')


Furthermore, SliDL makes it easy to apply a user-provided tissue detection model trained on additional, or alternative, annotated images by setting the modelStateDictPath and architecture parameters when calling Slide.detectTissue() to the path to the custom model and its neural network architecture, respectively.

**Code Listing 8**. **Apply a user-provided tissue detection model to a Slide**


slidl_slide.detectTissue(tissueDetectionLevel=1,
                            tissueDetectionTileSize=512,
                            tissueDetectionTileOverlap=0,
                            tissueDetectionUpsampleFactor=4,
                            batchSize=20,
                            numWorkers=16,
                            modelStateDictPath='/path/to/state_dict.pt',
                            architecture='vgg19')


In certain cases, users may want to make use of classical foreground filtering approaches in place of or in addition to the deep tissue detector. In SliDL, this can be achieved by calling the Slide.detectForeground() method, specifying the desired approach with the threshold argument and the desired WSI pyramid level to perform detection on with the level argument. Note that even if the foreground or deep tissue detector is applied at a different level from that which tiles are later extracted, the foreground/tissue/artefact/background predictions will be carried over appropriately across layers. SliDL currently supports Otsu’s method [19], the triangle algorithm [20], as well as simple intensity thresholding. All are automatically applied when Slide.detectForeground() so that the user is able to access the results of any algorithm in downstream functions.

**Code Listing 9**. **Apply foreground detection methods to a Slide**


slidl_slide.detectForeground(level=3)


In general, however, these approaches are less robust to the diversity of artefacts observed in WSIs, as well as appearances of background and tissue, often requiring careful supervision for each application [16]. We performed a benchmarking analysis comparing the performance of the deep tissue detector, Otsu’s and the Triangle algorithm at distinguishing tissue from background at the tile level (Fig 2B and 2C). Benchmarking was performed on 36 WSIs spanning 10 different tissues, a wide variety of different artefacts, and a range of different scanning and fixing protocols, in total comprising nearly 1.5 million tiles (see Benchmarking for full details). The balanced accuracy, sensitivity and specificity statistics indicate the strengths and weaknesses of each of the methods. The deep tissue detector has a significantly higher specificity to the Triangle algorithm, but is not significantly different from Otsu’s method, indicating that Otsu’s method is comparatively more conservative. The deep tissue detector has a significantly higher sensitivity to Otsu’s method, but is not significantly different from Triangle. Finally, the deep tissue detector has a significantly higher balanced accuracy to both methods, above 90%, indicating that while it does not decisively outperform both other methods in sensitivity and specificity individually, it does strike the best balance between the two. Furthermore, the deep tissue detector performs substantially more consistently at the task, indicating its greater robustness (Fig 2B). In Fig 2C we display three examples which are representative of the range of behaviours shown across the full set of slides.

### Annotation

Ground-truth labels for a WSI may exist either at the region-level, wherein they are local to particular regions within the WSI, or at the slide-level, wherein a label applies to the WSI as a whole. Region-level labels typically take the form of digital annotations on the WSI, delineating the regions belonging to certain classes. Specialised software such as QuPath and the Automated Slide Analysis Platform (ASAP) allow users to draw digital annotations onto WSIs and then export them for use in image analysis workflows [21, 22]. SliDL supports the use of annotation files in the GeoJSON format as well as the XML format output by ASAP [23]. QuPath annotations can be exported as GeoJSON files using the Groovy script, qupath_to_geojson.groovy provided in the SliDL tutorial repository (https://github.com/markowetzlab/slidl-tutorial).

An annotation file is added to a corresponding Slide object by calling the Slide.addAnnotations() method, providing the file path to the annotationFilePath argument. The annotations do not need to cover the entire WSI, instead SliDL parses annotations by designating all pixels bounded by an annotation for a given class as being positive for that class, and all other pixels as negative. Furthermore, in the case of annotations with ‘doughnut holes’ (annotations which are not polygons because they contain holes in their middle), users need to simply annotate the doughnut holes and assign them to their own class. Then, when calling Slide.addAnnotations(), the name of this class can be provided to the negativeClass argument. SliDL will automatically geometrically subtract these doughnut hole annotations from every other annotation class they overlap with. By default, Slide.addAnnotations() parses the annotations of all non-doughnut hole classes into the Slide object’s tile dictionary, but users can choose to include only certain classes present in the annotations by specifying them explicitly as a list to the classesToAdd argument.

**Code Listing 10**. **Add annotations to a Slide**


slidl_slide.addAnnotations(annotationFilePath='/path/to/annotations.xml',
                            classesToAdd=['normal', 'tumor'],
                            negativeClass='doughnut_holes',
                            level=0)


It is commonly the case that researchers wish to train their models exclusively on tiles which fall within annotated regions. As such, in SliDL tiles can be extracted from annotated regions by applying the Slide.extractAnnotationTiles() method. A simple heuristic is applied: a tile is extracted if there are any annotations that cover more than a given threshold fraction of the area of the tile. Tiles which are not covered above this threshold for any annotations are ignored entirely. The threshold is set using the tileAnnotationOverlapThreshold argument (default is 0.5). Different thresholds can be applied for different classes by providing a dictionary with class names as keys and their corresponding overlap thresholds as values to the tileAnnotationOverlapThreshold argument.

A user may also want to extract tissue tiles at random from a slide which has not been annotated. Calling Slide.extractRandomUnannotationTiles() achieves this, and involves most of the same arguments as Slide.extractAnnotationTiles(). The unannotatedClassName argument allows the user to specify how unannotated tiles should be named.

In addition, after calling the Slide.detectTissue() method on a given Slide object, each tile will be inferred on the deep tissue detector, and the resulting tissue probabilities will be stored for each tile in the ‘tile dictionary’. The tissueLevelThreshold argument can then be used in subsequent functions such as the tile extraction functions (Slide.extractAnnotationTiles() and Slide.extractRandomUnannotatedTiles()) to set a minimum tissue probability for a tile to be extracted (recommended value of 0.995).

Likewise, foregroundLevelThreshold can be used to restrict extraction of tiles to those reaching a desired foreground detection threshold as determined by foreground detection techniques such as [19] (set the argument to ‘otsu’) or the triangle algorithm [20] (set the argument to ‘triangle’). Simple average greyscale intensity filtering can be achieved by setting foregroundLevelThreshold to an integer between 0 and 100.

**Code Listing 11**. **Extract random unannotated tiles from a Slide’s WSI**


channel_data = slidl_slide.extractRandomUnannotatedTiles(
                            outputDir=output_dir,
                            numTilesToExtract= 500,
                            unannotatedClassName='tissue',
                            tissueLevelThreshold=0.995,
                            foregroundLevelThreshold=88)


Whether classifying or segmenting, it is important to consider how to derive the ground-truth labels for individual tiles from slide label data. For segmentation tasks the principle is straightforward because the ground-truth is at the pixel-level and thus whole-slide segmentation masks can also be tiled and directly inherited by the individual image tiles. As such, once annotations have been added, SliDL allows the user to create both binary and multi-class tile-level segmentation masks for provided annotations. When applying the Slide.extractAnnotationTiles() method, binary segmentation masks are created by setting the extractSegmentationMasks argument to True, and the classesToExtract argument set to the name of the class (or list of classes) for which the binary segmentation masks should be created. If the user wishes for empty class directories to be created for classes not present in the annotation, they can be defined with the otherClassNames argument. The tile-level masks are then saved to a ‘masks’ directory in the location specified by the outputDir argument of Slide.extractAnnotationTiles().

**Code Listing 12**. **Extract tiles from the annotated regions of a Slide’s WSI**


channel_data = slidl_slide.extractAnnotationTiles(
                            outputDir='/path/to/folder',
                            classesToExtract=['normal', 'tumor']
                            tileAnnotationOverlapThreshold=0.3,
                            numTilesToExtractPerClass=500,
                            extractSegmentationMasks=True,
                            tissueLevelThreshold=0.995,
                            foregroundLevelThreshold=88)


In the case of binary segmentation, a separate segmentation mask for each desired class will be extracted. In multi-class segmentation problems, users may want to return ‘stacks’ of tile segmentation masks, where each layer of the stack is the segmentation mask of a different class. SliDL allows users to easily generate these ‘stacked’ multi-class segmentation masks for each tile they extract. Slide.extractAnnotationTilesMultiClassSegmentation() performs the same basic tasks as Slide.extractAnnotationTiles(), extracting tiles and their corresponding segmentation masks from an annotated WSI, but instead of outputting flat segmentation mask images to the segmentation mask directories, it outputs stacked Numpy ndarray matrices (saved as .npy files) as multi-class segmentation masks for each tile instead.

**Code Listing 13**. **Extract segmentation mask tiles from the annotated regions of a Slide’s WSI**


channel_data = slidl_slide.extractAnnotationTilesMultiClassSegmentation(
                            outputDir=output_dir,
                            classesToExtract=['normal', 'tumor']
                            tileAnnotationOverlapThreshold=0.3,
                            numTilesToExtractPerClass=500,
                            tissueLevelThreshold=0.995,
                            foregroundLevelThreshold=88)


For classification tasks a tile inherits the label of any annotations that cover more than the given threshold fraction of the area of the tile specified by the tileAnnotationOverlapThreshold argument of the Slide.extractAnnotationTiles() method discussed above. In addition, this heuristic is also applied for the numTilesToExtractPerClass argument which allows the user to set the maximum number of tiles to extract per class from the slide (default is 100 for each class). This ensures that the user does not extract many more tiles than they need for training for a given class. When there are more extractable tiles for a given class than are requested by the user, numTilesToExtractPerClass tiles are selected at random from the class. This argument can be used regardless of whether the user is performing a classification or segmentation task downstream of the tile extraction.

In addition to extracting the tiles to directories, unless returnTileStats is set to False, Slide.extractAnnotationTiles() returns some summary statistics of all the tile images which were extracted. These values can be used to compute the mean and variance of each of the colour channels across the tile dataset, which some users may want to use to normalise their tiles prior to training.

### Training

Building a dataset with labels that can be interpreted by a deep learning library is an important training consideration. SliDL’s tile extraction functions (Slide.extractAnnotationTiles() and Slide.extractRandomUnannotatedTiles()) output tiles and labels in a directory structures that are by default compliant for direct input into PyTorch’s torchvision.datasets.ImageFolder dataset constructor (see S1 Fig), making it straightforward to load the tiles SliDL has extracted into a format ready for training.

For classification tasks, per torchvision.datasets.ImageFolder, tile labels are stored as directory names within a parent directory containing the slide or case name [12]. As SliDL has been designed to perform pre- and post-processing, training should be performed by using a separate, complementary library (see Related methods). During inference the inference step (Inference, SliDL is capable of accepting any PyTorch image model which has been trained, which includes both convolutional neural networks (CNNs) and vision transformers (ViTs).

#### Convolutional neural networks

The first of the two main types of deep neural networks used for image data today, and the sort of deep neural network used to train the deep tissue detector (Deep tissue detector) is the convolutional neural network, which has shown outstanding performance on image classification tasks [24–26]. CNNs are a type of learning algorithm designed to take as input data which are spatially invariant (also known as “shift invariant”), a characteristic whereby small translations of the input data are tolerated. Since recognising common patterns in image data benefits from this trait, CNNs trained on images have proven to be highly successful and are one of the main deep learning architectures used in computer vision, including on tasks involving WSIs [16, 25, 27].

**Fig 3:**
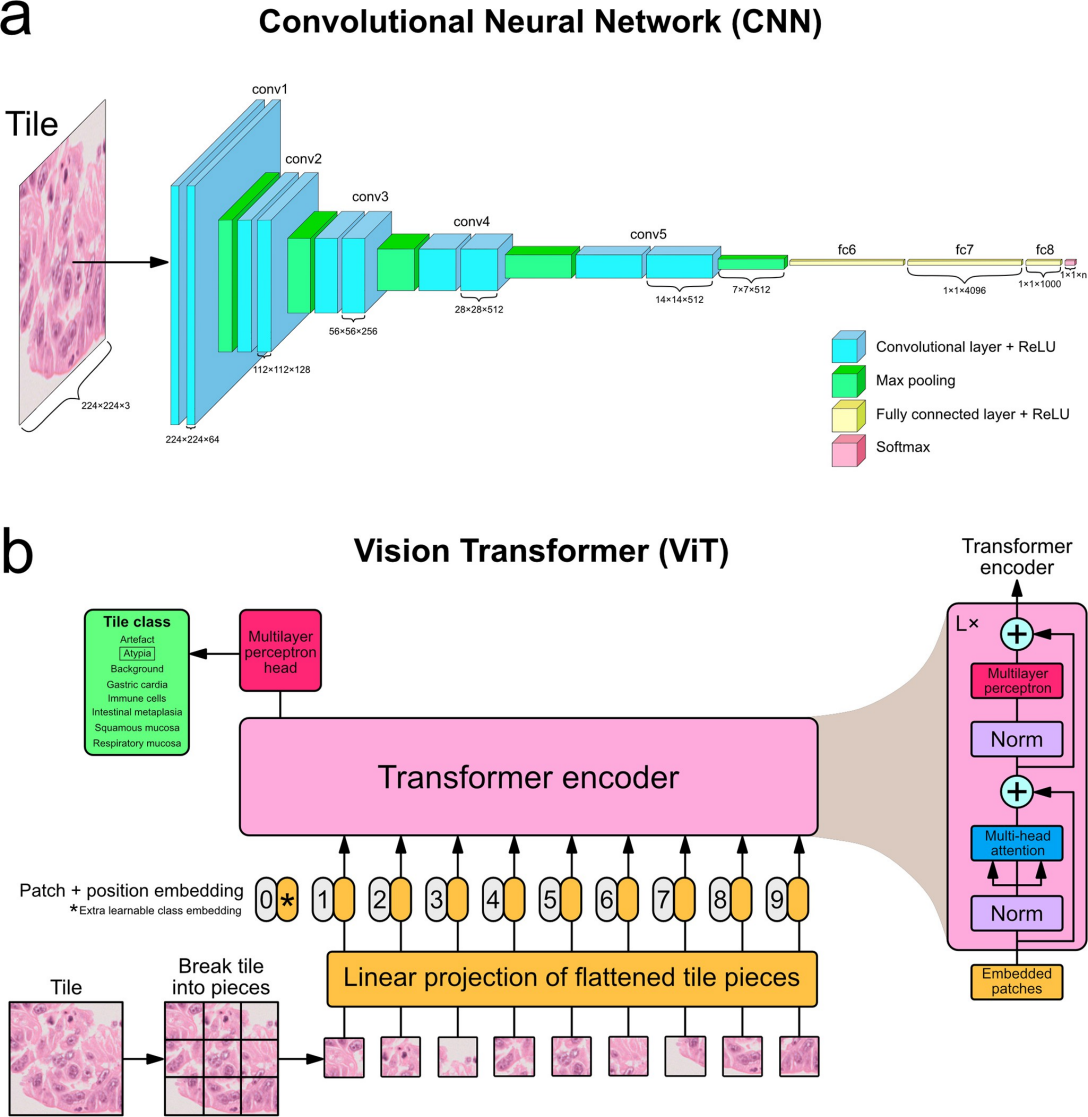
Structure of convolutional neural network and vision transformer. **(a)** A VGG-like convolutional neural network with alternating convolutional layers and maximum pooling operations before a few fully connected layers at the end. Figure is based on the VGG paper [29] and images of this architecture made by others. **(b)** A vision transformer, based on the figure in the original ViT paper [9].

Image-oriented CNNs work by including a set of two-dimensional convolution operations performed in a sweeping motion over the surface of the input image at each layer to transform it into an increasingly abstract representation (Fig 3). The weights in the filters (also known as the “kernels”) of each convolutional layer are what are used in these operations. The filter weights are learned during backpropagation, so that relevant aspects of the image discerned during training can be retained as the feature maps derived from the image are passed from one layer to the next. In between convolution layers are maximum pooling layers, which select the largest element within each receptive field to shrink the feature maps’ spatial resolution Fig 3. Through this reduction, pooling layers allow for the spatial invariance characteristic described above [28]. After the alternating convolutional and pooling layers are typically one or several fully-connected layers to coerce the number of features down to the desired class size (in the case of classification models) or down to the pixel mask size (in the case of segmentation models) [28].

CNNs have been the state of the art for classification and segmentatioon tasks on whole-slide images for several years [1].

#### Vision transformers

Transformers are a newer neural network type which was originally designed for sequence-based problems such as natural language processing (NLP), which is the use of machine learning to interpret text [8]. Previously, NLP tasks were primarily performed with recurrent neural networks (RNNs), but a fundamental limitation of RNNs is that the way they process sequences precludes computation from being parallelised during training, thereby limiting the length of sequences that can be trained on [8]. Attention mechanisms began to be used in RNNs to allow longer distances between sequence dependencies to be learned [30, 31]. Vaswani et al. [8] developed the first transformer architecture, which is composed entirely of attention mechanisms, entirely sidestepping the sequence dependency issue inherent to RNN-based models; transformers therefore tend to outperform and have functionally replaced RNNs [8, 32].

Dosovitskiy et al. [9] described the Vision Transformer (ViT), a transformer architecture modified to take image data as input. Although several CNNs had previously been designed to incorporate some attention mechanisms within them [33–35], this was the first successful architecture to train on images and use entirely attention mechanisms internally. This was achieved by splitting up inputted images into constituent pieces and treating these pieces (referred to as “tokens” in NLP language) as a sequence (Fig 3b). Their ViT model achieves state-of-the-art performance on several image classification benchmark datasets and has become a mainstay option for researchers looking for deep learning models trainable on images [9, 36–38].

Researchers have more recently begun applying vision transformers to whole-slide images and histopathological problems, but they form a promising new avenue [11].

### Inference

After a model has been trained, the next step is applying that model to the WSIs in a validation or test set to check the model’s performance. This application of a trained model to a new slide is known as inference. SliDL has two functions that infer a trained model on tiles with a sufficiently high tissue-probability (identified using the deep tissue detector) in a Slide object, saving the results into the tile dictionary internal to it: Slide.inferClassifier() and Slide.inferSegmenter(), which take as input a trained PyTorch model file. Using them is as simple as creating a Slide object for the slides one wants to infer on and then applying the function.

**Code Listing 14**. **Infer a classification or a segmentation model on a Slide’s WSI**


slidl_slide.inferClassifier(trainedModel=trained_classification_model,
                                classNames=['normal', 'tumor'],
                                dataTransforms=data_transforms,
                                tissueLevelThreshold=0.995,
                                foregroundLevelThreshold=88,
                                batchSize=30,
                                numWorkers=16)

slidl_slide.inferSegmenter(trainedModel=trained_segmentation_model,
                                classNames=['normal', 'tumor'],
                                dataTransforms=data_transforms,
                                tissueLevelThreshold=0.995,
                                foregroundLevelThreshold=88,
                                batchSize=30,
                                numWorkers=16)


After applying these functions, SliDL stores the predictions of the neural network for each tile in the tile dictionary internal to the Slide object.

**Fig 4:**
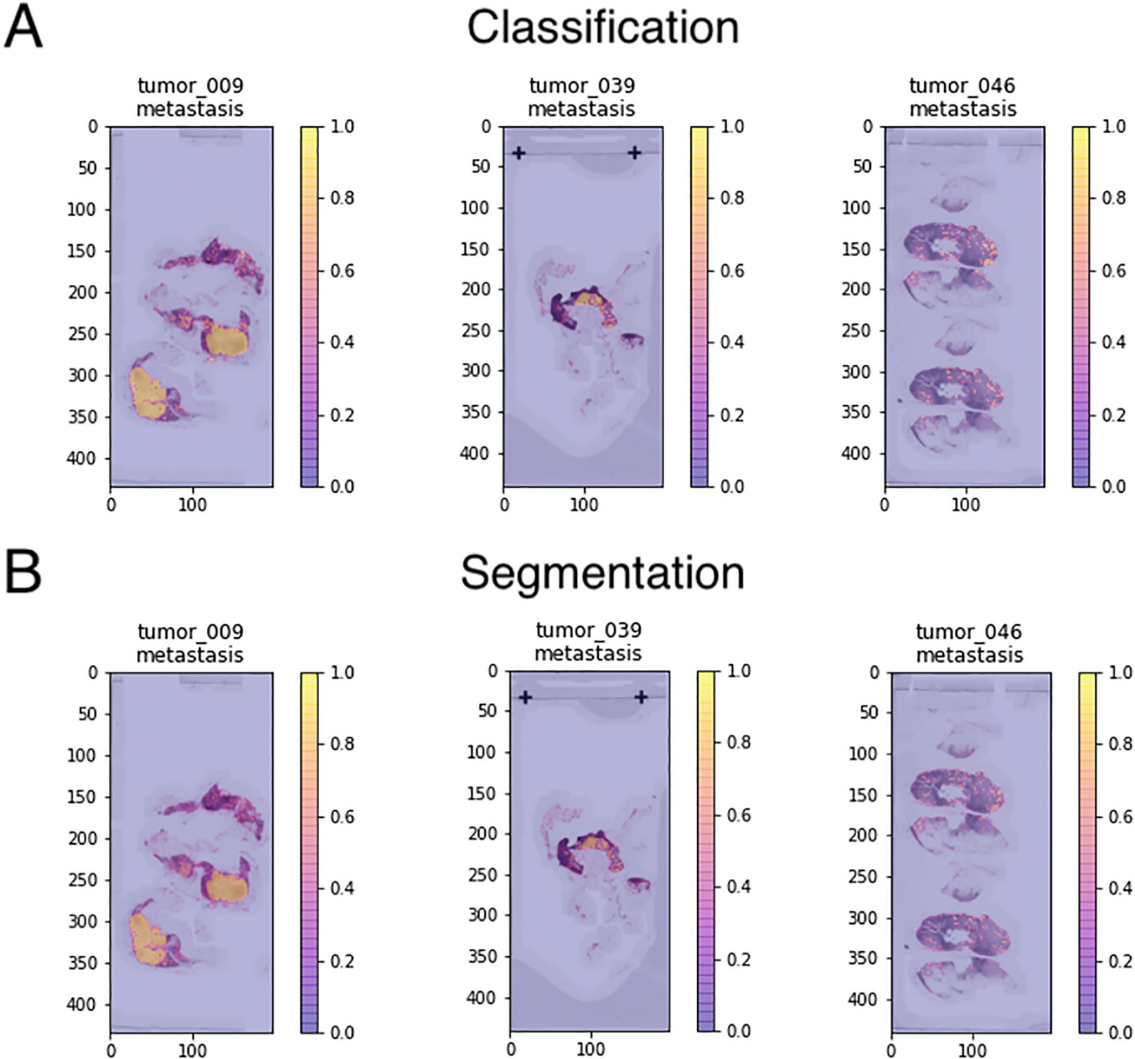
Visualising the inference of trained models. **(A)** A plot of the inference of a trained tile-level classification model on three validation slides from the SliDL tutorial, showing the ability of the classification model to distinguish regions showing breast cancer metastasis from normal lymph node tissue. Plots were generated with Slide.visualizeClassifierInference(). **(B)** A plot of the inference of a trained tile-level segmentation model on three validation slides from the SliDL tutorial, showing the ability of the segmentation model to distinguish the same regions as the classification model. Plots were generated with Slide.visualizeSegmenterInference().

To ensure that the model is behaving as expected, it is important to visualise inference results by plotting the inference predictions of tiles spatially as they appear in the WSI. Once inference has been performed on the Slide objects, SliDL’s Slide.visualizeClassifierInference() and Slide.visualizeSegmenterInference() functions create these plots for the user overlaid on a low-resolution image of the WSI, taking the class and WSI pyramid level to visualise as arguments. The user is therefore able to qualitatively assess whether the regions highlighted by the model are as expected, given the ground-truth, and experiment with different training configurations, tile sizes, or other hyperparameters before proceeding if not (Fig 4).

**Code Listing 15**. **Visualise the inference of a classification or a segmentation model on a Slide’s WSI**


slidl_slide.visualizeClassifierInference(classToVisualize='tumor',
                                            folder='path/to/folder',
                                            level=3)

slidl_slide.visualizeSegmenterInference(classToVisualize='tumor',
                                            folder='path/to/folder',
                                            level=3)


When performing segmentation, the user may want to create segmentation masks which are larger than the size of the tiles that were inferred on. When adjacent tiles overlap, tile-level segmentation masks cannot simply be concatenated. SliDL supports the automatic generation of whole-slide segmentation masks with the Slide.getNonOverlappingSegmentationInferenceArray() method, returning an inference array with the model’s pixel-level predictions and merging overlapping regions to return single predictions for each pixel. The class for which predictions are desired is specified by the className argument. Inference matrices are saved as compressed .npz files at the location specified by the folder argument. Users have the option of defining a threshold with the probabilityTheshold argument to return a binary output matrix, where pixels with a probability for the given class with a probability at or above the threshold are binarized to true, and the others to false. If the users does not define a probabilityTheshold, the raw probability value will be scaled to be between 0 and 255 (255 is 100% probability) and returned as a Numpy uint8 integer if the dtype argument is set to ‘int’ (the default). The user can also choose to have the probabilities returned as Numpy float32 floats if dtype is set to ‘float’, but this is usually undesirable as it results in extremely memory-intensive matrices.

**Code Listing 16**. **Save the segmentation matrix from an inference on a Slide’s WSI**


slidl_slide.getNonOverlappingSegmentationInferenceArray(
                                        className='tumor',
                                        dtype='int'
                                        folder='path/to/folder')


### Evaluating model performance

Beyond visual verification, numerical methods are requires to assess model performance. SliDL includes a number of wrapper functions around common performance metrics to enable easy model evaluation. After inferring using a trained model, the Slide.classifierMetricAtThreshold() and Slide.segmenterMetricAtThreshold() methods can be applied. By providing a list of probability thresholds to the probabilityThresholds argument, a metric (‘accuracy’, ‘balanced_accuracy’, ‘f1’, ‘precision’, or ‘recall’ for Slide.classifierMetricAtThreshold(), and ‘dice_coeff’ for segmenterMetricAtThreshold()), and a class to the classToThreshold argument, these methods will calculate the corresponding metric at each probability threshold for the given class.

For Slide.classifierMetricAtThreshold(), the tileAnnotationOverlapThreshold argument defines the minimum fraction of a tile’s area that must be covered by an annotation from the classToThreshold for that tile to be considered ground-truth positive for that class (default is 0.5).

**Code Listing 17**. **Compute the classification or segmentation accuracy of an inference on a Slide’s WSI**


classification_accuracies = slidl_slide.classifierMetricAtThreshold(
                            classToThreshold='tumor',
                            probabilityThresholds=probability_thresholds,
                            tileAnnotationOverlapThreshold=0.3,
                            metric='accuracy')

segmentation_accuracies = slidl_slide.segmenterMetricAtThreshold(
                            classToThreshold='tumor',
                            probabilityThresholds=probability_thresholds)


The threshold that gives best performance on the validation set can then be applied to the test set with the same two functions; inputting the single best threshold in the probablityThresholds argument.

Although inference is performed on the tile-level, when applied in the clinic a label for the entire slide is often required. Similarly, during training, it is frequently the case that the test set has only a slide-level label. A method is therefore needed to translate tile-level predictions to slide-level labels. One approach is to determine a threshold for the number of tiles which need to be called positive, for a given class at a given probability threshold, in order to call an entire slide positive. To test the performance of a model on data with only slide-level labels, the AUC for a ROC curve can be computed for a range of positive-tile counts. The optimum count can then be used when applying the best model to unlabelled data in the clinic. In order to make such analyses straightforward, SliDL includes the Slide.numTilesAboveClassPredictionThreshold() method which returns the number of tiles in a slide whose inference prediction probabilities for a given class (classToThreshold) are greater than or equal to given probability thresholds (provided in a list to the probablityThresholds argument).

**Code Listing 18**. **Count the number of tiles in a Slide whose inference value exceeds a certain threshold**


slidl_slide.numTilesAboveClassPredictionThreshold(
                            classToThreshold='tumor',
                            probabilityThresholds=probability_thresholds)


These positive-tile counts per slide can then be used to calculate an AUROC. As above, often a range of probability thresholds are tried, and the one which yields the largest AUROC on the validation set is then applied to the test set to give the final performance.

## Discussion

SliDL provides an easily-installable (see Installation) Python library with a straightforward API for users seeking to perform tasks involving artefact and background detection, tile extraction from annotation, and model training and inference on WSIs. It is intended for users with basic Python knowledge; it does not demand extensive experience with WSI data or pathology. We see this as one of SliDL’s key advantages over existing methods which expect more rigorous backgrounds in histopathology or image analysis. To best understand the unique value provided by SliDL, one must compare it to existing computation methods in the WSI space.

### Related methods

Several tools have been developed which also provide support for some of the functionalities available in SliDL. While providing a complete description and comparison of these tools is beyond the scope of this manuscript, here we briefly describe these tools and direct the reader towards the articles presenting them for more details (see S3 Table for a summary of all of the comparisons made with all the methods discussed below).

HistoQC [39] is a Python-based tool for performing quality control of WSIs, aiding users in the identification slides containing potential technical artefacts and affected by batch effects. By providing the user with modules for performing a wide range of classical image analysis techniques, HistoQC enables the construction of custom pipelines for performing foreground filtering, detection of slide artefacts such as pen marks, and identification of batch effects such as slides with darker staining compared to the rest. In HistoQC, this is achieved using a combination of approaches including inspection of colour distributions, application of edge and smoothness detectors, and classical filters such as Gabor and Frangi filters for texture analysis. For example, if the background of a WSI is uniformly white, foreground filtering can be performed by applying a threshold to the colour distribution which excludes white pixels. Similarly, a bright green pen mark may be clearly distinguishable from tissue by inspection of the green colour distribution of the WSI. In addition, HistoQC provides an interactive user interface for exploring one’s data. These approaches can achieve competitive results when carefully tuned by the user, but may struggle in more complex cases, such as uneven background, and pen marks with similar colour to the tissue. HistoQC is therefore a useful tool, complementing the wider functionality and robustness of SliDL, and enabling rapid quality control processing of one’s data.

HistomicsTK [40] is a Python library for performing a number of image analysis tasks specific to WSIs including stain colour deconvolution, normalisation and augmentation, as well as cell/nuclei segmentation and even a user interface for manual annotation of WSIs. Like HistoQC, all image analysis techniques are performed using classical approaches. HistomicsTK is highly complementary to SliDL, and in particular, we envisage that users may make use of HistomicsTK for performing WSI-specific colour augmentations within a SliDL workflow.

Histolab [41] is a Python library combining features found both in HistoQC and HistomicsTK, including functions for performing classical image analysis techniques to facilitate tissue detection and artefact removal, cell/nuclei segmentation, and colour transformations such as colour deconvolution. In addition, Histolab, like SliDL, supports the extracting of tiles from WSIs, and enables one to easily test alternative tiling strategies, including random extraction of tiles according to tissue detection score thresholds.

MONAI [42] is an extensive Python library which is part of the PyTorch ecosystem and is designed as a unified framework for performing deep learning on medical imaging data. Like SliDL, MONAI supports tiling of WSIs and provides extensive support model evaluation metrics. In addition, MONAI provides domain-specific support for data augmentation transforms, implementations of neural network architectures, optimisers, loss functions, and AI-assisted annotation, all of which are tuned for application to medical imaging data.

PathML [43] is a Python library which also supports the tiling of WSIs, and similar to Histolab, HistomicsTK and HistoQC implements an number of classical approaches to foreground and artefact detection. Similarly to MONAI and HistomicsTK, PathML also supports some pre-processing methods such as stain normalisation data augmentation, but is not designed to perform any post-processing steps.

### Distinct advantages of SliDL

Part of the advantage of SliDL is the ease with which it can be learned and used to render complicated digital histopathological techniques and problems immediately accessible to researchers, turning what would be weeks of work and files full of code into one straightforward, linear workflow consisting of just a few lines.

In addition to these advantages, the SliDL toolbox we present here improves on the other tools mentioned above in several ways, most importantly the handling of digital annotations. None of the tools listed above implement deep tissue detectors, nor do they implement tools for handling of annotations for WSIs to facilitate labelling of tiles, automatic resolution of annotation conflicts, or generation of binary and multi-class tile-level segmentation masks. Furthermore, while all of these tools provide support for the pre-processing of WSIs, only MONAI provides tools for model evaluation, and none support post-processing tasks such as the stitching together of tile-level segmentation masks to produce a slide-level mask. Tools such as MONAI are therefore highly complementary to SliDL: we envisage users, for example, making use of SliDL for pre-processing and handling of annotations, and MONAI for data augmentation and implementation of neural network architectures for training.

### Future work

Despite its many advantages, SliDL can be expanded upon and improved in various ways. For example, it might be useful to eventually provide a graphical user interface (GUI) to SliDL so that some of its basic features are accessible to users with less of a computer science background. This might include sliders, toggles, and text boxes in place of programmatic functions with arguments.

Furthermore, currently, applying the deep tissue detector with any efficiency to WSIs requires a computer vision-grade GPU, ideally with at least 4 gigabytes of memory and a GPU clock of at least 1200 megahertz. This feature will therefore be slow for users working on most standard commercial laptop or desktop models. For this reason, it is worth exploring alternative methods of implementing SliDL’s deep tissue detector which are compatible with lower-specification hardware. One potential solution is to re-train the deep tissue detector model on a deep neural network with fewer internal parameters, such as MnasNet [44], SqueezeNet [45], or MobileNet [46].

Finally, there remain many potential features to add to SliDL to expand its breadth. We would like to include a function to automatically generate tile annotations files demarcating the highest probability tiles for a certain class identified during inference. We are also interested in including functions so that SliDL can generate of saliency maps of trained deep learning models [47].

## Conclusion

SliDL is a new and fully-functional new tool for computer scientists looking for a straightforward but powerful Python library for solving some of the most commonly faced and nettlesome problems for training and evaluating deep learning models on WSI data quickly and easily. SliDL also includes a fully illustrative tutorial Jupyter notebook on a real-world example problem on a publicly available dataset from beginning (removing background and slide artefacts, extracting tiles from annotations) to end (training a deep learning model, inferring it on new slides, and evaluating model performance); the tutorial can be found here: https://slidl.readthedocs.io/en/latest/. It is our hope and goal that with SliDL and its corresponding tutorial, the application of deep learning to whole-slide images becomes more accessible not just to researchers already involved in digital pathology, but to newcomers as well, making the field on the whole more approachable.

## Availability and requirements

**Project name**: SliDL

**Project home page**: https://github.com/markowetzlab/slidl

**Project tutorial home page**: https://github.com/markowetzlab/slidl-tutorial

**Project documentation home page**: https://slidl.readthedocs.io/en/latest/

**Operating system(s)**: Not applicable

**Programming language**: Python (version 3.7 or above)

**Other requirements**: Not applicable

**License**: GPL-3.0

**Any restrictions to use by non-academics**: Not applicable

## Materials and installation

### Benchmarking

Here is a full breakdown of the slides used for the benchmarking analysis of the deep tissue detector (see Deep tissue detector):

- 36 slides
- 6 TCGA-TGCT (testicular germ cell tumor) H&E
- 6 CAMELYON-16 (tiny breast cancer metastases in lymph nodes, including slides with and without metastases present) H&E slides
- 6 OCCAMS (esophageal adenocarcinoma) H&E slides from esophago-gastro-duodenoscopy
- 6 BEST2 (cytosponge samples some with Barett’s) P53 and H&E slides
- 6 TCGA-PRAD (prostate adenocarcinoma) H&E slides
- 2 TCGA floor of mouth cancer H&E slides
- 1 TCGA small intestine cancer H&E slide
- 1 TCGA gum cancer H&E slide
- 1 TCGA “spinal cord, cranial nerves, and other unspecified parts of central nervous system” cancer H&E slide
- 1 TCGA tonsil cancer H&E slide
- Mixture of samples preserved via FFPE (formalin fixed paraffin embedded) and those preserved via flash freezing
- Different scanning file types, scanning machines, times of scanning, scanning locations, stain intensities, stains (H&E and P53), tissue removal methods/surgery types, and tissue types
- Slide artefacts included ink of many different colors (including blue, red, black, and reen—some used to mark regions on top of tissue, other times to write labels on a background portion of a slide), slide bubbles, tissue folding/burning, cloudiness or yellowing, slide shifted in scanning machine to leave black region in image, slide edge artefacts, dirt and debris under the slide, black slide crosses, blurry regions
- Slides exhibited a wide range of artefact degree and amount—some slides had virtually no artefact whatsoever, others had a few little ink dots or bubbles, still others had large black regions and/or regions of ink etc.
- 1,489,084 tiles in total across all 36 slides with a massive range in the size of each slide (smallest slide: 47MB, largest slide: 3.9GB / fewest tiles in one slide: 3,304, most: 86,190)
- 730 individual annotations including tissue and doughnut hole annotations
- Slides all scanned in 40x with 500px edge length tiles

All 36 WSIs used to benchmark the deep tissue detector are available at https://doi.org/10.5281/zenodo.7947380.

### Hardware

It is recommended that SliDL users either work on a machine with at least 32 GB of RAM and enough disk space to hold the number of number of tiles they would like to extract (tiles are not large, but if thousands are extracted, a proportional amount of disk space is required. At least four cores are recommended. WSIs tend to be 0.5–5 GB in size, so if tens or hundreds are used in an analysis, disk space to store them is required (external hard drives work well). Users that wish to utilise the inference functions of Slide, Slide.inferClassifier() and Slide.inferSegmenter() are highly recommended to have a CUDA-compatible Graphics Processing Unit (GPU). SliDL also works well in high performance computing environments that meet these conditions.

### Installation

SliDL is available on the Python Package Index (PyPI) for easy installation:

**Code Listing 19**. Install SliDL


pip install slidl


### Troubleshooting

There are several mistakes and error messages that can arise when using SliDL. Troubleshooting presents the most common mistakes users might run into, including the error message output by SliDL, the possible reason for the mistake, and the possible solution to it (Table 1).

**Table 1:** Common SliDL error messages with explanations and possible solutions. *X*, *Y*, and *Z* represent numbers or words that will vary depending on the exact error made by the user.

Message	Possible reason	Solution
‘This image is not compatible. Please refer to the documentation for proper installation of openslide and libvips’	The WSI input into the SliDL initialiser was not a supported file format	Convert the WSI to a file format supported by lipvips.
‘Tissue detection has already been performed. Use overwriteExistingTissueDetection if you wish to write over it’	Slide.detectTissue() has already been called on the Slide object	Set the overwriteExistingTissueDetection argument to True, or else don’t perform tissue detection again
‘Annotation with centroid (*X*, *Y*) produces a Shapely *Z* instead of a polygon; check to see if it self-intersects.’	The annotation around the specified centroid pixel coordinates of the WSI does not produce a polygon when geometrically parsed	Check that annotation on the WSI in a WSI viewer looking for self-overlapping regions and correct it to be a polygon
‘Warning: *X* suitable *Y* tiles found but requested *Z* tiles to extract. Extracting all suitable tiles…’	The numTilesToExtractPerClass argument of a tile extraction function exceeds the number of suitable tiles of class *Y*	Reduce the numTilesToExtractPerClass argument for class *Y*, or else let SliDL will extract all available *Y* tiles by default
‘Model has *X* classes but only Y class names were provided in the classes argument’	The number of classes output by the model inputted to Slide.inferClassifier() or Slide.inferSegmenter() does not equal the number of classes present in the classNames argument	Verify that classNames includes all the classes that were trained on, and correct this argument as necessary
‘No predictions found in slide. Use inferClassifier() / inferSegmenter() to generate them.’	Using a SliDL function reliant on inference results without having added inference results to the Slide object	Run Slide.inferClassifier or Slide.inferSegmenter() on the Slide object

## Supporting information

S1 Fig Directory structure. The directory structure output by Slide.extractAnnotationTiles() and Slide.extractRandomUnannotatedTiles() which is amenable to PyTorch’s ImageFolder dataset.
(TIF)

S1 Table Table summarising the primary functions of SliDL. (TIF)

S2 Table Table describing the SliDL functions discussed in the main text. Functions are listed in green, a summary of their purpose in blue, their arguments in yellow and the description of the arguments in white. For a complete description of all SliDL functions and their arguments, see https://slidl.readthedocs.io/.
(PDF)

S3 Table Table of comparisons to related methods. (PDF)

## Abbreviations

API: Application Public Interface
BEST2: Barrett’s oEsophagus Screening Trial 2 [48]
OCCAMS: Oesophageal Cancer Clinical and Molecular Stratification [49]
TCGA: The Cancer Genome Atlas [50]
WSI: Whole-Slide Image

## References

[1] Dimitriou N, Arandjelović O, Caie PD. Deep Learning for Whole Slide Image Analysis: An Overview. Frontiers in medicine. 2019;6:264. doi: 10.3389/fmed.2019.00264 31824952 PMC6882930

[2] Kather JN, Heij LR, Grabsch HI, Loeffler C, Echle A, Muti HS, et al. Pan-cancer image-based detection of clinically actionable genetic alterations. Nature cancer. 2020;1(8):789–799. doi: 10.1038/s43018-020-0087-6 33763651 PMC7610412

[3] Lu MY, Chen TY, Mahmood F, et al. AI-based pathology predicts origins for cancers of unknown primary. Nature. 2021;594:106–110. doi: 10.1038/s41586-021-03512-4 33953404

[4] Montgomery E. Is there a way for pathologists to decrease interobserver variability in the diagnosis of dysplasia? Archives of pathology & laboratory medicine. 2005;129(2):174–176. doi: 10.5858/2005-129-174-ITAWFP 15679414

[5] Raab SS, Grzybicki DM. Anatomic pathology workload and error; 2006. 10.1309/YYL4-BK3C-BXP6-MCR8 16690476

[6] Goodfellow I, Pouget-Abadie J, Mirza M, Xu B, Warde-Farley D, Ozair S, et al. Generative adversarial networks. Communications of the ACM. 2020;63(11):139–144. doi: 10.1145/3422622

[7] Prakash CD, Karam LJ. It GAN DO better: GAN-based detection of objects on images with varying quality. IEEE Transactions on Image Processing. 2021;30:9220–9230. doi: 10.1109/TIP.2021.3124155 34735343

[8] Vaswani A, Shazeer N, Parmar N, Uszkoreit J, Jones L, Gomez AN, et al. Attention is all you need. Advances in neural information processing systems. 2017;30.

[9] Dosovitskiy A, Beyer L, Kolesnikov A, Weissenborn D, Zhai X, Unterthiner T, et al. An image is worth 16x16 words: Transformers for image recognition at scale. arXiv preprint arXiv:201011929. 2020.

[10] Jose L, Liu S, Russo C, Nadort A, Di Ieva A. Generative adversarial networks in digital pathology and histopathological image processing: A review. Journal of Pathology Informatics. 2021;12(1):43. doi: 10.4103/jpi.jpi_103_20 34881098 PMC8609288

[11] Zheng Y, Gindra RH, Green EJ, Burks EJ, Betke M, Beane JE, et al. A graph-transformer for whole slide image classification. IEEE transactions on medical imaging. 2022;41(11):3003–3015. doi: 10.1109/TMI.2022.3176598 35594209 PMC9670036

[12] Paszke A, Gross S, Massa F, Lerer A, Bradbury J, Chanan G, et al. PyTorch: An Imperative Style, High-Performance Deep Learning Library. In: Advances in Neural Information Processing Systems 32. Curran Associates, Inc.; 2019. p. 8024–8035. Available from: http://papers.neurips.cc/paper/9015-pytorch-an-imperative-style-high-performance-deep-learning-library.pdf.

[13] Litjens G, Bandi P, Bejnordi BE, Van der Laak J. 1399 H&E-stained sentinel lymph node sections of breast cancer patients: the CAMELYON dataset. GigaScience. 2018;7(6). doi: 10.1093/gigascience/giy065 29860392 PMC6007545

[14] Medixant. RadiAnt DICOM Viewer; 2021. Available from: https://www.radiantviewer.com.

[15] Martinez K, Cupitt J. VIPS—a highly tuned image processing software architecture. In: Proceedings of IEEE International Conference on Image Processing; 2005. p. 574–577.

[16] Janowczyk A, Madabhushi A. Deep learning for digital pathology image analysis: A comprehensive tutorial with selected use cases. J Pathol Inform. 2016;7(29). doi: 10.4103/2153-3539.186902 27563488 PMC4977982

[17] Huang G, Liu Z, Van Der Maaten L, Weinberger KQ. Densely connected convolutional networks. In: Proceedings of the IEEE conference on computer vision and pattern recognition; 2017. p. 4700–4708.

[18] Yang M. A PyTorch imbalanced dataset sampler for oversampling low frequent classes and undersampling high frequent ones; 2018–2020. https://github.com/ufoym/imbalanced-dataset-sampler.

[19] Otsu N. A threshold selection method from gray-level histograms. IEEE transactions on systems, man, and cybernetics. 1979;9(1):62–66. doi: 10.1109/TSMC.1979.4310076

[20] Zack GW, Rogers WE, Latt SA. Automatic measurement of sister chromatid exchange frequency. J Histochem Cytochem. 1977;25(7):741–753. doi: 10.1177/25.7.70454 70454

[21] Bankhead P, Loughrey MB, Fernández JA. QuPath: Open source software for digital pathology image analysis. Sci Rep. 2017;7:16878. doi: 10.1038/s41598-017-17204-5 29203879 PMC5715110

[22] Computation Pathology Group, part of the Diagnostic Image Analysis Group, at the Radboud University Medical Center. The Automated Slide Analysis Platform (ASAP); 2018. Available from: https://github.com/computationalpathologygroup/ASAP.

[23] Chamberlain S, Ooms J. geojson: Classes for ‘GeoJSON’; 2023.

[24] LeCun Y, Boser B, Denker JS, Henderson D, Howard RE, Hubbard W, et al. Backpropagation applied to handwritten zip code recognition. Neural computation. 1989;1(4):541–551. doi: 10.1162/neco.1989.1.4.541

[25] LeCun Y, Bottou L, Bengio Y, Haffner P. Gradient-based learning applied to document recognition. In: Proceedings of the IEEE; 1998. p. 2278–2324.

[26] Miotto R, Wang F, Wang S, Jiang X, Dudley JT. Deep learning for healthcare: review, opportunities and challenges. Briefings in bioinformatics. 2018;19(6):1236–1246. doi: 10.1093/bib/bbx044 28481991 PMC6455466

[27] Krizhevsky A, Sutskever I, Hinton GE. Imagenet classification with deep convolutional neural networks. In: Advances in Neural Information Processing Systems; 2012. p. 1097–1105.

[28] Rawat W, Wang Z. Deep Convolutional Neural Networks for Image Classification: A Comprehensive Review. Neural Computation. 2017;29(9):2352–2449. doi: 10.1162/neco_a_00990 28599112

[29] Simonyan K, Zisserman A. Very deep convolutional networks for large-scale image recognition. arXiv preprint arXiv:14091556. 2014.

[30] Bahdanau D, Cho K, Bengio Y. Neural machine translation by jointly learning to align and translate. arXiv preprint arXiv:14090473. 2014.

[31] Kim Y, Denton C, Hoang L, Rush AM. Structured attention networks. arXiv preprint arXiv:170200887. 2017.

[32] Lin T, Wang Y, Liu X, Qiu X. A survey of transformers. arXiv preprint arXiv:210604554. 2021.

[33] Wang X, Girshick R, Gupta A, He K. Non-local neural networks. In: Proceedings of the IEEE conference on computer vision and pattern recognition; 2018. p. 7794–7803.

[34] Carion N, Massa F, Synnaeve G, Usunier N, Kirillov A, Zagoruyko S. End-to-end object detection with transformers. In: European conference on computer vision. Springer; 2020. p. 213–229.

[35] Ramachandran P, Parmar N, Vaswani A, Bello I, Levskaya A, Shlens J. Stand-alone self-attention in vision models. Advances in Neural Information Processing Systems. 2019;32.

[36] Liu Y, Zhang Y, Wang Y, Hou F, Yuan J, Tian J, et al. A survey of visual transformers. arXiv preprint arXiv:211106091. 2021.

[37] Khan S, Naseer M, Hayat M, Zamir SW, Khan FS, Shah M. Transformers in vision: A survey. ACM computing surveys (CSUR). 2022;54(10s):1–41. doi: 10.1145/3505244

[38] Han K, Wang Y, Chen H, Chen X, Guo J, Liu Z, et al. A survey on vision transformer. IEEE transactions on pattern analysis and machine intelligence. 2022. 35180075 10.1109/TPAMI.2022.3152247

[39] Janowczyk A, Zuo R, Gilmore H, Feldman M, Madabhushi A. Automatic Measurement of Sister Chromatid Exchange Frequency. JCO Clin Cancer Inform. 2019;3:1–7. 10.1200/CCI.18.00157 PMC6552675 30990737

[40] Gutman DA, Khalilia M, Lee S, Nalisnik M, Mullen Z, Beezley J, et al. The Digital Slide Archive: A Software Platform for Management, Integration and Analysis of Histology for Cancer Research. Cancer research. 2018;77(21):e75–e78. doi: 10.1158/0008-5472.CAN-17-0629 PMC5898232 29092945

[41] Marcolini A, Bussola N, Arbitrio E, Amgad M, Jurman G, Furlanello C. histolab: A Python library for reproducible Digital Pathology preprocessing with automated testing. SoftwareX. 2022;20:101237. doi: 10.1016/j.softx.2022.101237

[42] MONAI Consortium. MONAI: Medical Open Network for AI; 2020. Available from: https://github.com/Project-MONAI/MONAI.

[43] Rosenthal J, Carelli R, Omar M, Brundage D, Halbert E, Nyman J, et al. Building Tools for Machine Learning and Artificial Intelligence in Cancer Research: Best Practices and a Case Study with the PathML Toolkit for Computational Pathology. Molecular Cancer Research. 2022;20(2):202–206. doi: 10.1158/1541-7786.MCR-21-0665 34880124 PMC9127877

[44] Tan M, Chen B, Pang R, Vasudevan V, Sandler M, Howard A, et al. Mnasnet: Platform-aware neural architecture search for mobile. In: Proceedings of the IEEE/CVF Conference on Computer Vision and Pattern Recognition; 2019. p. 2820–2828.

[45] Iandola FN, Han S, Moskewicz MW, Ashraf K, Dally WJ, Keutzer K. SqueezeNet: AlexNet-level accuracy with 50x fewer parameters and< 0.5 MB model size. arXiv preprint arXiv:160207360. 2016.

[46] Howard AG, Zhu M, Chen B, Kalenichenko D, Wang W, Weyand T, et al. Mobilenets: Efficient convolutional neural networks for mobile vision applications. arXiv preprint arXiv:170404861. 2017.

[47] Selvaraju RR, Cogswell M, Das A, Vedantam R, Parikh D, Batra D. Grad-CAM: Visual explanations from deep networks via gradient-based localization. In: Proceedings of the IEEE International Conference on Computer Vision; 2017. p. 618–626.

[48] Ross-Innes C, et al. Risk stratification of Barrett’s oesophagus using a non-endoscopic sampling method coupled with a biomarker panel: a cohort study. Lancet Gastroenterol Hepatol. 2017;2(1):23–31. doi: 10.1016/S2468-1253(16)30118-2 28404010

[49] Peters CJ, Rees JR, Hardwick RH, Hardwick JS, Vowler SL, Ong CAJ, et al. A 4-gene signature predicts survival of patients with resected adenocarcinoma of the esophagus, junction, and gastric cardia. Gastroenterology. 2010;139(6):1995–2004. doi: 10.1053/j.gastro.2010.05.080 20621683

[50] Cancer Genome Atlas Research Network, Weinstein JN, Collisson EA, Mills GB, Shaw KR, Ozenberger BA, et al. The Cancer Genome Atlas Pan-Cancer analysis project. Nat Genet. 2013;45(10):1113–1120. doi: 10.1038/ng.2764 24071849 PMC3919969

