# Supplementary figures and images for: SliDL: A toolbox for processing whole-slide images in deep learning
Source: PLoS One. 2023 Aug 7;18(8):e0289499. doi: 10.1371/journal.pone.0289499 (PMC10406329; doi:10.1371/journal.pone.0289499)

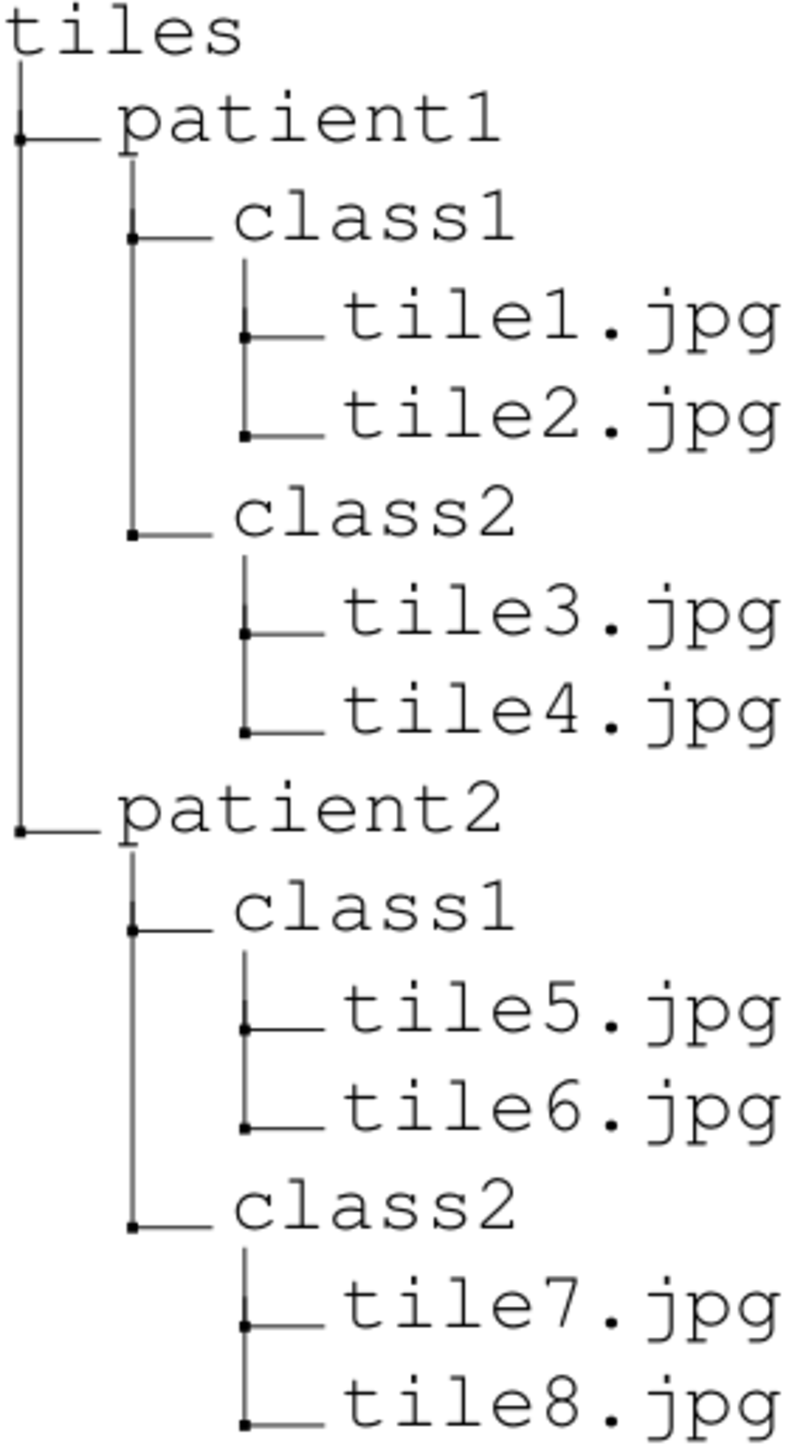

Supplement: S1 Fig — The directory structure output by Slide.extractAnnotationTiles() and Slide.extractRandomUnannotatedTiles() which is amenable to PyTorch’s ImageFolder dataset. (TIF) [file pone.0289499.s001.tif]

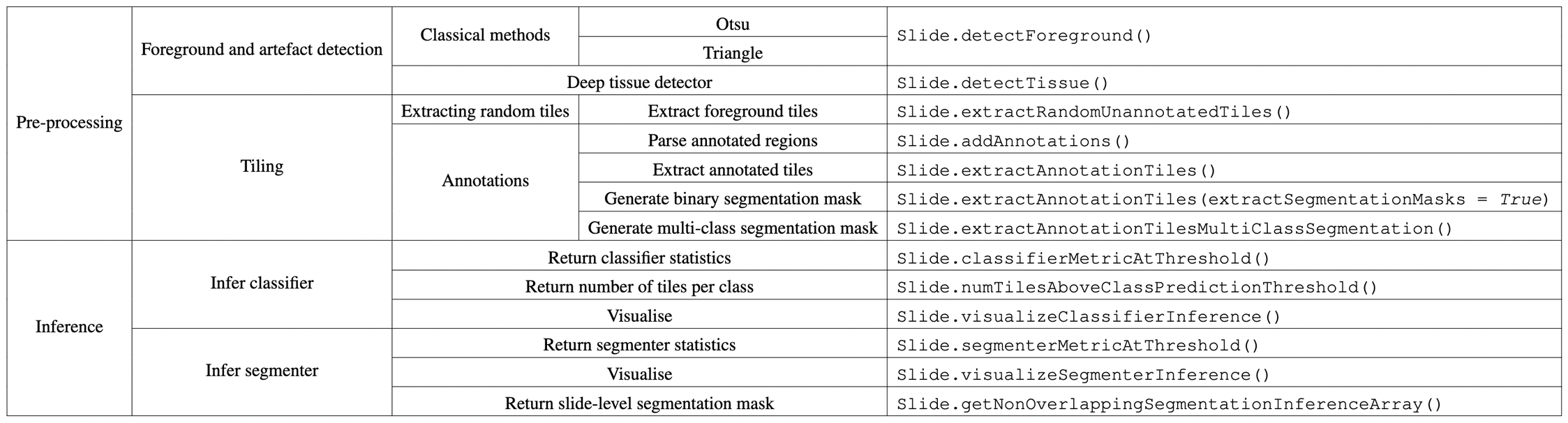

Supplement: S1 Table — (TIF) [file pone.0289499.s002.tif]
