# Supplementary material for: SliDL: A toolbox for processing whole-slide images in deep learning
Source: PLoS One. 2023 Aug 7;18(8):e0289499. doi: 10.1371/journal.pone.0289499 (PMC10406329; doi:10.1371/journal.pone.0289499)
Supplement: S2 Table — Functions are listed in green, a summary of their purpose in blue, their arguments in yellow and the description of the arguments in white. For a complete description of all SliDL functions and their arguments, see https://slidl.readthedocs.io/. (PDF) [file pone.0289499.s003.pdf]

`slidl.slide.Slide(slideFilePath, newSlideFilePath=False, level=0, verbose=False)`	
The main class of SliDL; a representation of whole-slide image containing the dictionary of tiles, and upon which further analyses are added, including but not limited to tissue detection and annotation, and from which tiles from whole-slide images can be extracted.	
`slideFilePath` (str)	path to a WSI (to make from scratch) or to a .pml file
`level` (int, optional)	the level of the WSI pyramid at which to operate on; 0 is the highest resolution and default and how many levels are present above that depends on the WSI
`Slide.setTileProperties(self, tileSize, tileOverlap=0, unit='px')`	
A function to set the properties of the tile dictionary in a Slide object. Should be the first function called on a newly created Slide object.	
`tileSize` (int)	the edge length of each square tile in the requested unit
`tileOverlap` (float, optional)	the fraction of a tile's edge length that overlaps the left, right, above, and below tiles. Default is 0.
`unit` (str, optional)	the unit to measure tileSize by. Default is 'px' for pixels and no other units are current supported
`Slide.iterateTiles(self, tileDictionary=False, includeImage=False, writeToNumpy=False)`	
A generator function to iterate over all tiles in the tile dictionary, returning the tile address or the tile address and the tile image if specified with includeImage.	
`Slide.detectTissue(self, tissueDetectionLevel=1, tissueDetectionTileSize=512, tissueDetectionTileOverlap=0, tissueDetectionUpsampleFactor=4, batchSize=20, numWorkers=16, overwriteExistingTissueDetection=False, modelStateDictPath='../pathml/pathml/models/deep-tissue-detector_densenet_state-dict.pt', architecture='densenet')`	

A function to apply SliDL's built-in deep tissue detector to assign artefact, background, and tissue probabilities that sum to one to each tile in the tile dictionary. The raw tissue detection map for a WSI is saved into a Slide attribute called rawTissueDetectionMap in the Slide which can be loaded into a new Slide object to save inference time with `Slide.detectTissueFromRawTissueDetectionMap()`. For this reason, calling `Slide.save()` after `Slide.detectTissue()` finishes is recommended.	
`tissueDetectionLevel` (int, optional)	the level of the WSI pyramid at which to perform the tissue detection. Default is 1.
`tissueDetectionTileSize` (int, optional)	the edge length in pixels of the tiles that the deep tissue detector will be inferred on. Default is 512.
`tissueDetectionTileOverlap` (float, optional)	the fraction of a tile's edge length that overlaps the left, right, above, and below tiles. Default is 0.
`tissueDetectionUpsampleFactor` (int, optional)	the factor why which the WSI should be upsampled when performing tissue detection. Default is 4.
`batchSize` (int, optional)	the number of tiles per minibatch when inferring on the deep tissue detector. Default is 20.
`numWorkers` (int, optional)	the number of workers to use when detecting tissue. Default is 16.
`modelStateDictPath` (str, optional)	the path to the state dictionary of the deep tissue detector; it must be a 3-class classifier, with the class order as follows: background, artifact, tissue. Default is the path to the state dict of the deep tissue detector build into SliDL.
`architecture` (str, optional)	the name of the architecture that the state dict belongs to. Currently supported architectures include resnet18, inceptionv3, vgg16, vgg16_bn, vgg19, vgg19_bn, densenet, alexnet, and squeezenet. Default is "densenet", which is the architecture of SliDL's built-in deep tissue detector.
`Slide.visualizeTissueDetection(self, fileName=`*`False`*`, folder=os.getcwd())`	
A function to generate a 3-color tissue detection map showing where on a WSI the deep tissue detector applied with `Slide.detectTissue()` artefact was found (red), where background was found (green), and where tissue was found (blue). The resulting image is saved at the following path: folder/fileName/fileName_tissuedetection.png	

<table>
<tr><td colspan="2">Slide.detectForeground(self, level=4, overwriteExistingForegroundDetection=*False*, threshold=None)</td></tr>
<tr><td colspan="2">A function to implement traditional foreground filtering methods on the tile dictionary to exclude background tiles from subsequent operations.</td></tr>
<tr><td>level (int, optional)</td><td>the level of the WSI pyramid to detect foreground on. Default is 4. Not all WSIs will have a 4th level, so alter if necessary. If memory runs out, increase the level to detect foreground with a less high resolution image.</td></tr>
<tr><td colspan="2">Slide.addAnnotations(self, annotationFilePath, classesToAdd=*False*, negativeClass=*False*, level=0, overwriteExistingAnnotations=*False*, mergeOverlappingAnnotationsOfSameClass=*True*, acceptMultiPolygonAnnotations=*True*)</td></tr>
<tr><td colspan="2">A function that adds the overlap between all (desired) classes present in an annotation file and each tile in the tile dictionary Annotations within groups in ASAP are taken to be within one class, where the name of the ASAP group is the name of the class; similarly, annotations within classes in QuPath are taken to be within one class, where the name of the QuPath class is the name of the class. (except the negativeClass if one is specified). Acceptable ASAP annotation tools to make annotations for this function include the RectangleAnnotation, PolyAnnotation, and SplineAnnotation tools; in QuPath, acceptable tools include the Rectangle, Ellipse, Polygon, and Brush tools. Annotations should be polygons, i.e. closed regions that do not self-overlap at any point. Annotations of different classes are expected to never overlap, and annotations of the same class can only overlap (and will be merged into one polygon) if mergeOverlappingAnnotationsOfSameClass is set to *True*.</td></tr>
<tr><td>annotationFilePath (str)</td><td>the path to the file containing the annotation. The file must be either an xml file from the ASAP software or a GeoJSON file from the QuPath software.</td></tr>
<tr><td>classesToAdd (list of str, optional)</td><td>a list of classes to add from the annotation file. Default is that all annotation classes will be used.</td></tr>
<tr><td>negativeClass (str, optional)</td><td>the name of the class of negative annotations (donut holes) to subtract from the other annotations. Default is not to consider any class to be a negative space class.</td></tr>
<tr><td>level (int, optional)</td><td>the level of the WSI pyramid to make use of. Default is 0.</td></tr>
<tr><td colspan="2">Slide.extractRandomUnannotatedTiles(self, outputDir, slideName=*False*, numTilesToExtract=100, unannotatedClassName='unannotated', otherClassNames=*False*, extractSegmentationMasks=*False*, foregroundLevelThreshold=*False*, tissueLevelThreshold=*False*, returnTileStats=*True*, seed=*False*)</td></tr>
</table>

A function to extract randomly selected tiles that don't overlap any annotations into directory structure amenable to `torch.utils.data.ConcatDataset`	
`outputDir` (str)	the path to the directory where the tile directory will be stored
`numTilesToExtract` (int, optional)	the number of random unannotated tiles to extract. Default is 50.
`unannotatedClassName` (str, optional)	the name that the unannotated "class" directory should be called. Default is "unannotated".
`tissueLevelThreshold` (Bool, optional)	if defined, only extracts tiles with a 0 to 1 tissueLevel probability greater than or equal to the set value. Default is False.
`foregroundLevelThreshold` (str or int or float, optional)	if defined as an int, only extracts tiles with a 0-100 foregroundLevel value less or equal to than the set value (0 is a black tile, 100 is a white tile). Only includes Otsu's method-passing tiles if set to 'otsu', or triangle algorithm-passing tiles if set to 'triangle'. Default is not to filter on foreground at all.
`Slide.extractAnnotationTiles(self, outputDir, slideName=False, numTilesToExtractPerClass='all', classesToExtract=False, otherClassNames=False, extractSegmentationMasks=False, tileAnnotationOverlapThreshold=0.5, foregroundLevelThreshold=False, tissueLevelThreshold=False, returnTileStats=True, returnOnlyNumTilesFromThisClass=False, seed=False)`	
A function to extract tiles that overlap with annotations into directory structure amenable to `torch.utils.data.ConcatDataset`.	
`outputDir` (str)	the path to the directory where the tile directory will be stored
`classesToExtract` (str or list of str, optional)	defaults to extracting all classes found in the annotations, but if defined, must be a string or a list of strings of class names.
`tileAnnotationOverlapThreshold` (float, optional)	a number greater than 0 and less than or equal to 1, or a dictionary of such values, with a key for each class to extract. The numbers specify the minimum fraction of a tile's area that overlaps a given class's annotations for it to be extracted. Default is 0.5.

<table>
<tr><td><code>numTilesToExtractPerClass</code> (dict or int or 'all', optional)</td><td>how many suitable tiles to extract from the slide for each class; if more suitable tiles are available than are requested, tiles will be chosen at random; expected to be positive integer, a dictionary with class names as keys and positive integers as values, or ‘all’ to extract all suitable tiles for each class. Default is ‘all’.</td></tr>
<tr><td><code>extractSegmentationMasks</code> (Bool, optional)</td><td>whether to extract a ‘masks’ directory that is exactly parallel to the ‘tiles’ directory, and contains binary segmentation mask tiles for each class desired. Pixel values of 255 in these masks appear as white and indicate the presence of the class; pixel values of 0 appear as black and indicate the absence of the class. Default is False.</td></tr>
<tr><td><code>tissueLevelThreshold</code> (Bool, optional)</td><td>if defined, only extracts tiles with a 0 to 1 tissueLevel probability greater than or equal to the set value. Default is False.</td></tr>
<tr><td><code>foregroundLevelThreshold</code> (str or int or float, optional)</td><td>if defined as an int, only extracts tiles with a 0-100 foregroundLevel value less or equal to than the set value (0 is a black tile, 100 is a white tile). Only includes Otsu’s method-passing tiles if set to ‘otsu’, or triangle algorithm-passing tiles if set to ‘triangle’. Default is not to filter on foreground at all.</td></tr>
<tr><td colspan="2"><code>Slide.extractAnnotationTilesMultiClassSegmentation(self, outputDir, slideName=False, numTilesToExtract=100, classesToExtract=False, tileAnnotationOverlapThreshold=0.5, foregroundLevelThreshold=False, tissueLevelThreshold=False, returnTileStats=True, seed=False)</code></td></tr>
<tr><td colspan="2">A function to extract tiles that overlap with annotations and their corresponding segmentation masks, where annotation masks are returned as .npy files containing ndarray stacks (each array in the stack being one class’s segmentation class) for use in multi-class segmentation problems.</td></tr>
<tr><td><code>outputDir</code> (str)</td><td>the path to the directory where the tile directory will be stored</td></tr>
<tr><td><code>classesToExtract</code> (str or list of str, optional)</td><td>defaults to extracting all classes found in the annotations, but if defined, must be a string or a list of strings of class names.</td></tr>
</table>

`tileAnnotationOverlapThreshold` (float, optional)	a number greater than 0 and less than or equal to 1, or a dictionary of such values, with a key for each class to extract. The numbers specify the minimum fraction of a tile's area that overlaps a given class's annotations for it to be extracted. Default is 0.5.
`numTilesToExtractPerClass` (dict or int or 'all', optional)	how many suitable tiles to extract from the slide for each class; if more suitable tiles are available than are requested, tiles will be chosen at random; expected to be positive integer, a dictionary with class names as keys and positive integers as values, or 'all' to extract all suitable tiles for each class. Default is 'all'.
`extractSegmentationMasks` (Bool, optional)	whether to extract a 'masks' directory that is exactly parallel to the 'tiles' directory, and contains binary segmentation mask tiles for each class desired. Pixel values of 255 in these masks appear as white and indicate the presence of the class; pixel values of 0 appear as black and indicate the absence of the class. Default is False.
`tissueLevelThreshold` (Bool, optional)	if defined, only extracts tiles with a 0 to 1 tissueLevel probability greater than or equal to the set value. Default is False.
`foregroundLevelThreshold` (str or int or float, optional)	if defined as an int, only extracts tiles with a 0-100 foregroundLevel value less or equal to than the set value (0 is a black tile, 100 is a white tile). Only includes Otsu's method-passing tiles if set to 'otsu', or triangle algorithm-passing tiles if set to 'triangle'. Default is not to filter on foreground at all.
`Slide.inferClassifier(self, trainedModel, classNames, dataTransforms=None, batchSize=30, numWorkers=16, foregroundLevelThreshold=`*`False`*`, tissueLevelThreshold=`*`False`*`, overwriteExistingClassifications=`*`False`*`)`	
A function to infer a trained classifier on a Slide object using PyTorch.	
`trainedModel` (torchvision.models)	A PyTorch torchvision model that has been trained for the classification task desired for inference.
`classNames` (list of str)	an alphabetized list of class names.

`dataTransforms` (torchvision.transforms.Compose)	a PyTorch torchvision.Compose object with the desired data transformations.
`foregroundLevelThreshold` (str or int or float, optional)	if defined as an int, only infers trainedModel on tiles with a 0-100 foregroundLevel value less or equal to than the set value (0 is a black tile, 100 is a white tile). Only infers on Otsu's method-passing tiles if set to 'otsu', or triangle algorithm-passing tiles if set to 'triangle'. Default is not to filter on foreground at all.
`tissueLevelThreshold` (Bool, optional)	if defined, only infers trainedModel on tiles with a 0 to 1 tissueLevel probability greater than or equal to the set value. Default is False.
`batchSize` (int, optional)	the number of tiles to use in each inference minibatch.
`numWorkers` (int, optional)	the number of workers to use when inferring the model on the WSI. Default is 16.
`Slide.inferSegmenter(self, trainedModel, classNames, dataTransforms=None, dtype='int', batchSize=1, numWorkers=16, foregroundLevelThreshold=False, tissueLevelThreshold=False, overwriteExistingSegmentations=False)`	
A function to infer a trained segmentation model on a Slide object using PyTorch.	
`trainedModel` (torchvision.models)	A PyTorch segmentation model that has been trained for the segmentation task desired for inference.
`classNames` (list of str)	a list of class names. The first class name is expected to correspond with the first channel of the output mask image, the second with the second, and so on.
`dataTransforms` (torchvision.transforms.Compose)	a PyTorch `torchvision.Compose` object with the desired data transformations.
`foregroundLevelThreshold` (str or int or float, optional)	if defined as an int, only infers trainedModel on tiles with a 0-100 foregroundLevel value less or equal to than the set value (0 is a black tile, 100 is a white tile). Only infers on Otsu's method-passing tiles if set to 'otsu', or triangle algorithm-passing tiles if set to 'triangle'. Default is not to filter on foreground at all.

`tissueLevelThreshold` (Bool, optional)	if defined, only infers trainedModel on tiles with a 0 to 1 tissueLevel probability greater than or equal to the set value. Default is `False`.
`batchSize` (int, optional)	the number of tiles to use in each inference minibatch.
`numWorkers` (int, optional)	the number of workers to use when inferring the model on the WSI. Default is 16.
`Slide.visualizeClassifierInference(self, classToVisualize, fileName=`*`False`*`, folder=os.getcwd(), level=4)`	
A function to create an inference map image of a Slide after running `Slide.inferClassifier()` on it. The resulting image is saved at the following path: folder/fileName/fileName_classification_of_classToVisualize.png	
`classToVisualize` (str)	the class to make an inference map image for. This class must be present in the tile dictionary from `Slide.inferClassifier()`.
`folder` (str, optional)	the path to the directory where the map will be saved. Default is the current working directory.
`level` (int, optional)	the level of the WSI pyramid to make the inference map image from.
`Slide.visualizeSegmenterInference(self, classToVisualize, probabilityThreshold=None, fileName=`*`False`*`, folder=os.getcwd(), level=4)`	
A function to create an inference map image of a Slide after runnin `Slide.inferSegmenter()` on it. Tiles are shown with the averageof the probabilities of all their pixels. To get a pixel-level probability matrix, use `Slide.getNonOverlappingSegmentationInferenceArray()`. The resulting image is saved at the following path: /folder/fileName/fileName_segmentation_of_classToVisualize.png	
`classToVisualize` (str)	the class to make an inference map image for. This class must be present in the tile dictionary from `Slide.inferSegmenter()`.
`folder` (str, optional)	the path to the directory where the map will be saved. Default is the current working directory.
`level` (int, optional)	the level of the WSI pyramid to make the inference map image from.

`Slide.getNonOverlappingSegmentationInferenceArray(self, className, aggregationMethod='mean', probabilityThreshold=None, dtype='int', folder=os.getcwd(), verbose=False)`	
A function to extract the pixel-wise inference result (from `Slide.inferSegmenter()`) of a Slide. Tile overlap is "stitched together" to produce one mask with the same pixel dimensions as the WSI. The resulting mask will be saved to a .npz file as a scipy.sparse.lil_matrix.	
`className` (str)	the name of the class to extract the binary mask for. Must be present in the tile dictionary from `Slide.inferSegmenter()`.
`dtype` (str, optional)	the data type to store in the output matrix. Options are 'int' for numpy.uint8 (the default), 'float' for numpy.float32. To get a Boolean output using a probability threshold, set a value for probabilityThreshold.
`folder` (str, optional)	the path to the directory where the scipy.sparse.lil_matrix will be saved. Default is the current working directory.
`Slide.classifierMetricAtThreshold(self, classToThreshold, probabilityThresholds, tileAnnotationOverlapThreshold=0.5, metric='accuracy', assignZeroToTilesWithoutAnnotationOverlap=True)`	
A function to return the tile-level metric of a class probability threshold (or list of thresholds) compared to the ground truth, where a tile with ground truth annotation overlap greater than or equal to `tileAnnotationOverlapThreshold` is considered to be ground truth positive for that class. Ground truth annotations are expected to have been added to each tile in the tile dictionary by `Slide.addAnnotations()`. Class probability labels are expected to have been added to each tile in the tile dictionary by `Slide.inferClassifier()`. Metrics include 'accuracy', 'balanced_accuracy', 'f1', 'precision', or 'recall'.	
`classToThreshold` (str)	the class to threshold the tiles by. The class must be already present in the tile dictionary from `Slide.inferClassifier()`.

`probabilityThresholds` (float or list of floats)	the probability threshold or list of probability thresholds (in the range 0 to 1) to check. If a float is provided, just that probability threshold will be used, and a float of the accuracy of the classifier using that threshold as when the model considered a tile positive for the class will be returned. If a list of floats is provided, a list of floats of accuracies for those thresholds will be returned in respective order the inputted threshold list will be returned.
`tileAnnotationOverlapThreshold` (float, optional)	the class annotation overlap threshold at or above which a tile is considered ground truth positive for that class. Default is 0.5.
`metric` (str, optional)	which metric to compute. Options are ‘accuracy’, ‘balanced_accuracy’, ‘f1’, ‘precision’, or ‘recall’. Default is ‘accuracy’.
`Slide.segmenterMetricAtThreshold(self, classToThreshold, probabilityThresholds, metric='dice_coeff')`	
A function to return the pixel-level metric of a class probability threshold (or list of thresholds) compared to the ground truth, where a pixel with ground truth annotation overlap greater than or equal to `probabilityThresholds` is considered to be ground truth positive for that class. Ground truth annotations are expected to have been added to each tile in the tile dictionary by `Slide.addAnnotations()`. Class probability labels are expected to have been added to each tile in the tile dictionary by `Slide.inferSegmenter()`. The only metric currently available is the Dice coefficient. The metric will be applied to all tiles with predictions added by `Slide.inferSegmenter()` and the average of that metric across those tiles will be returned to give one metric per slide per threshold in `probabilityThresholds`.	
`classToThreshold` (str)	the class to threshold the pixels by. The class must be already present in the tile dictionary from `Slide.inferSegmenter()`.
`probabilityThresholds` (float or list of floats)	the probability threshold or list of probability thresholds (in the range 0 to 1) to check. If a float is provided, just that probability threshold will be used, and a float of the accuracy of the segmenter using that threshold as when the model considered a pixel positive for the class will be returned. If a list of floats is provided, a list of floats of accuracies for those thresholds will be returned in respective order the inputted threshold list will be returned.

Slide.numTilesAboveClassPredictionThreshold(self, classToThreshold, probabilityThresholds)	
A function to return the number of tiles at or above one or a list of probability thresholds for a classification class added to each tile in the tile dictionary by Slide.inferClassifier().	
classToThreshold (str)	the class to threshold the tiles by. The class must be already present in the tile dictionary from Slide.inferClassifier().
probabilityThresholds (float or list of floats)	the probability threshold or list of probability thresholds (in the range 0 to 1) to check. If a float is provided, just that probability threshold will be used, and an int of the number of tiles at or above that threshold will be returned. If a list of floats is provided, a list of ints of the number of tiles at or above each of those thresholds in respective order to the inputted threshold list will be returned.

S2 Table: **Table describing the SliDL functions discussed in the main text.** Functions are listed in green, a summary of their purpose in blue, their arguments in yellow and the description of the arguments in white. For a complete description of all SliDL functions and their arguments, see https://slidl.readthedocs.io/.
