# Supplementary material for: SliDL: A toolbox for processing whole-slide images in deep learning
Source: PLoS One. 2023 Aug 7;18(8):e0289499. doi: 10.1371/journal.pone.0289499 (PMC10406329; doi:10.1371/journal.pone.0289499)
Supplement: S3 Table — (PDF) [file pone.0289499.s004.pdf]

Task	SliDL	HistoQC	HistomicsTK	Histolab	MONAI	PathML
Tile extraction using manual annotation files	✓	x	✓	x	x	x
Handling of manual multiclass annotations for tile extraction	✓	x	x	x	✓	x
Ability to generate manual annotation within the software	x	x	✓	x	✓	x
Ability to resolve small annotation mistakes (loops, overlaps, etc.)	✓	x	✓	x	x	x
Parses doughnut-hole annotations appropriately	✓	x	✓	x	x	x
AI-assisted annotation within the software	x	x	x	x	✓	x
Extracts segmentation masks matching paired with tiles	✓	x	✓	✓	✓	✓
Classical tissue filtering (foreground filtering)	✓	✓	✓	✓	✓	✓
Complex artifact filtering	✓	✓	✓	✓	✓	✓
Fully automated complex artifact filtering	✓	x	x	x	x	x
Quality control - automatic detection of slides with unusual colour/texture/artefact properties	x	✓	x	x	x	x
Quality control - automatic detection of possible missing or repeated data	x	✓	x	x	x	x
Ability to perform inference on trained classification models	✓	x	x	x	✓	✓
Ability to perform inference on trained segmentation models	✓	x	x	x	✓	✓
Creates visual inference maps	✓	x	x	x	✓	✓

Exports inference results	✓	x	x	x	✓	✓
WSI specific image normalisation approaches	x	x	✓	✓	✓	✓
WSI specific data augmentation approaches	x	x	✓	✓	✓	✓
Performing training task within software generally speaking	x	x	x	x	✓	✓
In-built models for training with transfer learning	x	x	x	x	✓	x
In-built training schedulers/optimizers/loss functions	x	x	x	x	✓	✓

S3 Table: **Table of comparisons to related methods.**
